# Psychometric properties of persian version of escapism scale among Iranian adolescents

**DOI:** 10.1186/s40359-023-01379-w

**Published:** 2023-10-10

**Authors:** Roghieh Nooripour, Nikzad Ghanbari, Simin Hosseinian, Carl J. Lavie, Nazir Mozaffari, Sverker Sikström, Seyed Ruhollah Hosseini

**Affiliations:** 1 Department of Counseling, Faculty of Education and Psychology, Alzahra University, Tehran, Iran; 2 Faculty of Education and Psychology, Shahid Beheshti University, Tehran, Iran; 3 Department of Cardiovascular Diseases, Ochsner Clinical School, John Ochsner Heart and Vascular Institute, University of Queensland School of Medicine, New Orleans, LA USA; 4 Department of Psychology, Lund university, Lund, Sweden; 5 Department of Clinical Psychology, Faculty of Education and Psychology, Ferdowsi University of Mashhad, Mashhad, Iran

**Keywords:** Escapism, Identity, Adolescent, Reliability, Validity

## Abstract

The aim of this study was to assess the psychometric properties of the Persian version of the Escapism Scale among Iranian adolescents aged 14–18. Between January 2021 and August 2021, cross-sectional study was conducted using a convenience sampling method to select 566 participants (340 girls and 226 boys) to investigate the relationship between physical activity and mental health in adolescents. The participants completed several questionnaires, including the Escapism Scale, Erikson Psychosocial Stage Inventory (EPSI), Satisfaction with Life Scale (SWLS), Hope Scale (AHS), Smartphone Addiction Scale-Short Version (SAS-SV), and General Self-efficacy (GSE). Construct validity, reliability using Cronbach’s alpha, and concurrent validity were used to evaluate the Escapism Scale’s validity and reliability. Results of the Confirmatory Factor Analysis (CFA) indicated that a two-factor model provided a good fit for the data: sbX2 = 179.99 (p < 0.01); SRMR = 0.07; RMR = 0.56, CFI = 0.91; NFI = 0.89; IFI = 0.91; NFI = 0.89; GFI = 0.93; AGFI = 0.90, RMSEA = 0.076). The Cronbach’s alpha coefficient for escapism was 0.73. The study found a significant positive relationship between escapism and smartphone addiction (r = 0.19). Additionally, a significant negative relationship was observed between escapism and hope (r=-0.31), satisfaction with life (r=-0.34), and general self-efficacy (r=-0.33). Furthermore, a significant relationship was found between escapism and gender. Lastly, the study found a significant relationship between escapism and identity confusion (r = 0.164, P < 0.01) and identity coherence (P < 0.01, r = 29). In conclusion, the Escapism Scale is a valid and reliable tool for assessing escapism and psychological evaluations in Iranian adolescents. These results may inform future research and suggest re-testing in clinical populations.

## Introduction

The concept of the self has become a prominent topic in psychology in recent decades after a period of relative neglect [1]. Currently, the concept is viewed as a complex problem with far-reaching implications and applications. In fact, no other topic has received as much attention in psychology as the self [2]. However, some theorists argue that the definition of the self has become more complex due to the shift in focus, and none of the descriptions are generally accepted in terms of their goals, objectives, and characteristics [3]. The self plays a central role in regulating behavior, acting as an intermediary between an individual’s external and internal worlds. It includes their knowledge of personal characteristics and abilities, as well as their thoughts about themselves [4]. A social cognitive perspective suggests that the self can be divided into two domains: content and structure. In terms of content, individuals’ self-descriptions change as they age, shifting from physical and material characteristics to explanations of their activities and skills [5].

The concept of escapism refers to various types of freedom from daily life affairs [6], survival mechanisms [7], and coping with emotional pressures caused by stressful situations [8]. Escapism is a psychological state caused by task absorption, temporary detachment, and reduced self-evaluation. When an individual engages in an activity to escape from another task, they experience absorption in the new activity, temporary dissociation, and reduced self-evaluation. This can have negative effects on time management, goal orientation, self-regulation, and learning.

In academic and research literature, escapism is often defined as the act of engaging in activities excessively as a means of coping with difficult life situations [9]. While these activities can produce a positive psychological experience of flow [10], they can also result in self-destructive behaviors, such as eating disorders and suicidal thoughts [11–13]. Task absorption, temporary dissociation, and lowered self-evaluation are among the affordances associated with escapism [14]. Despite the psychological benefits that escapism can offer for various activities, it can also have both positive and negative psychological outcomes [15]. The outcomes of escapism depend on the motivations behind it [14]. According to the regulatory focus theory, escapism can be induced either by motives that lead to positive affect or by motives that prevent negative affect, depending on the motives that lead to the desire to escape [16]. Positive psychological outcomes can be achieved when the activity is focused on self-expansion rather than self-defense. When an activity involves prevention-self-suppression, positive self-evaluation and ruminations are temporarily blocked, but it may also negatively affect future positive experiences and affective outcomes [17, 18].

Numerous studies have investigated escapism and confirmed its fundamental assumptions. Empirical evidence shows that self-expansion and self-suppression are distinct [14]. Additionally, the measures used to distinguish these two concepts had different associations with them. Participation in activities that promote self-expansion was strongly linked to positive affective outcomes, but not to depression vulnerability [19] or emotion suppression as an emotion regulation strategy [17, 20]. Furthermore, escapism has not been found to be related to subjective well-being in previous studies [21]. Moreover, longitudinal studies conducted over three months have revealed that there is a correlation between self-suppression motives and general negative affect, while the opposite was observed for self-expansion. In challenging times, activity engagement increases due to the self-suppression motive, without affecting self-expansion. When escapism, flow, and positive affective outcomes were included in a path model, flow positively mediated escapism and positive affective outcomes, leading to a flow towards positive affective outcomes. Furthermore, the correlation between self-suppression and a lower flow rate and effectiveness was related to self-suppression, resulting in a negative affective outcome. Finally, cognitive experiences such as flow experiences may influence the motives for escapism. Longitudinal studies conducted over three months have indicated that there is a correlation between general negative affect and self-suppression motives, whereas self-expansion was not associated with negative affect [22].

During difficult times, individuals tend to engage in activities due to the self-suppression motive, without affecting self-expansion. Positive affective outcomes were found to positively mediate escapism and flow when included in a path model. However, the correlation between self-suppression and a lower flow rate and effectiveness was associated with self-suppression, leading to negative affective outcomes. It is suggested that cognitive experiences, such as flow experiences, may influence the motives for escapism [14].

It has been previously noted that activity engagement is supported by a dualistic form of escapism [14]. This means that individuals may engage in certain activities to escape daily demands or avoid critical self-evaluation, and their personality traits, emotions, and overall well-being can influence their motivation to participate in activities they consider valuable. In essence, people’s experiences with their preferred activities and other activities that define their identity may be shaped by their motivation. Thus, understanding the role of motivation in activity engagement can be critical in shaping psychological outcomes in both cognitive and affective domains [11]. Defining escapism scientifically remains a challenge. This concept pertains to individuals who engage in specific activities to escape daily demands or avoid critical self-evaluation [23]. A two-dimensional model of escapism shares three essential characteristics. Firstly, it examines people’s experiences with their favorite activities and other activities that define their identity. Secondly, it proposes that the two dimensions are continuous rather than categorical. The study concludes by suggesting that motivation significantly shapes psychological outcomes resulting from engagement, and there is evidence of positive results in both cognitive and affective domains [14].

Studies have indicated a significant correlation between escapism and identity styles, particularly that individuals with diffuse-avoidant and foreclosed identity styles tend to rely more on escapist behaviors to cope with stress and anxiety, whereas those with achieved and moratorium identity styles use more active and problem-focused coping mechanisms and therefore use less escapist behaviors [24]. These findings suggest that one’s identity style may play a role in their tendency to use escapism as a coping strategy. Escapism can offer a temporary respite from negative emotions, creating a sense of hope that things will improve. For instance, a study found that individuals who used video games as a form of escapism reported a sense of hope and optimism as a result. However, escapism can also lead to a lack of motivation to address problems or make positive changes, undermining feelings of hope [25]. Escapism can sometimes be employed to avoid or distract oneself from difficult or uncomfortable situations rather than facing and working through them. People who use excessive social media as a form of escapism have reported lower levels of hope and higher levels of anxiety and depression. Escapism can also provide a temporary escape from the stresses and challenges of everyday life, leading to short-term life satisfaction. For example, a study found that individuals who used fantasy sports as a form of escapism reported higher levels of life satisfaction [26]. However, excessive or problematic use of escapism can have negative effects on long-term life satisfaction. This is because escapism can sometimes be used as a way to avoid or distract oneself from real-world problems or responsibilities, leading to feelings of dissatisfaction and unfulfillment. A study showed that people who used problematic social media as a form of escapism reported lower levels of life satisfaction [27].Overall, the relationship between escapism and life satisfaction appears to be intricate and dependent on specific circumstances and motivations for engaging in escapism. While it can provide temporary satisfaction and relief, problematic use of escapism can have negative effects on long-term life satisfaction [28]. Smartphones can provide a convenient and accessible means of engaging in escapism, such as through social media or mobile gaming. This can lead to an increased likelihood of developing problematic or addictive behaviors related to smartphone use. For instance, some researchers have shown that problematic smartphone use was associated with higher levels of escapism [29]. Excessive or problematic use of smartphones for escapism can lead to negative consequences, such as social isolation, decreased productivity, and mental health issues, ultimately perpetuating and exacerbating smartphone addiction [30]. Furthermore, the impact of escapism on self-efficacy may depend on the nature and duration of the escapism, as well as individual differences in coping styles and personality traits. On the one hand, escapism can provide a temporary break from stressors and challenges, allowing individuals to recharge and gain a sense of control over their environment, leading to increased self-efficacy in the short-term. For example, students who used online gaming as a form of escapism reported higher levels of self-efficacy [25]. However, the relationship between escapism and self-efficacy appears to be intricate and may depend on the specific context and motivations for engaging in escapism. While it can provide temporary relief and increase self-efficacy in the short-term, excessive or problematic use of escapism can have negative effects on long-term self-efficacy. Generally, researchers have also found a pattern of associations for the existence of a relationship between escapism and eating disorders [31], gambling disorder [32], impulse buying [33], clinical depression [34], alcohol consumption [35], excessive online gaming [36], Facebook overuse/addiction [37] and suicide attempt [11].

Although escapism is a crucial concept in psychology, there has been a dearth of studies conducted on it, possibly due to the complexity and subjectivity of the phenomenon. The limited research on escapism raises concerns about the understanding and treatment of mental health issues that may be linked to excessive escapist behaviors. To fill this gap in the literature, we conducted a study to measure the escapism scale among Iranian adolescents, which had not been done before. The study aimed to assess the psychometric properties of the Persian escapism scale among this group using a sample of 566 participants selected through a convenience sampling method. The findings of this study could contribute to a better understanding of escapism in Iranian adolescents and provide a helpful tool for psychologists to assess and treat mental health issues related to excessive escapism.

## Methods

This study adopted a cross-sectional design and was conducted between January 2021 and August 2021.

### Participants

To enhance the methodological rigor of the study, we utilized the STROBE checklist for cross-sectional studies [38]. The statistical population consisted of all Iranian adolescents, from which 566 participants (340 girls and 226 boys) were selected through convenience sampling. The research questionnaires were administered online, using various platforms in Iran such as Instagram, Telegram, WhatsApp, internet ads, and email.

Participants were required to meet several criteria: (1) be enrolled in high school (approximately 14–18 years old) to align with Erik Erikson’s psychosocial theory of development [39], (2) be fluent in Farsi and Iranian citizens, (3) have no history of mental or cognitive disorders, (4) not have had surgery that prevented them from exercising, and (5) provide written consent. The study excluded individuals with mental health issues such as anxiety, stress, or undiagnosed depression [39]. To ensure the accuracy of participant responses, we recommended reviewing the questionnaire before answering. Additionally, we set required answers for each item and ensured anonymity of responses, with participants not being required to provide their names, and participation being voluntary.

### Procedure

The survey was conducted in compliance with ethical guidelines, with the consent of both the participants and their parents or legal guardians, and their privacy was strictly maintained. The participants were provided with informed consent and were invited to complete online questionnaires, which were distributed through social networks. The survey also collected demographic information, and participation was voluntary, anonymous, and coded for test-retest reliability. Respondents had the option to participate, provide information, or withdraw at any time. The reliability of the survey was assessed using Cohen’s kappa coefficient, which indicated an acceptable agreement between the two rates (K = 0.76). All procedures were conducted in accordance with the relevant guidelines and regulations. Additionally, in this study, we used other scales that had previously been translated into Farsi, in addition to the escapism scale.

The study was divided into three phases, namely translation, adaptation, and cultural adaptation, according to methodology of Brislin [40]. During the first phase, the Escapism Scale was translated into Farsi using a back-translation technique. This method involves translating the measurement into the target language by one translation team and then translating it back into the source language by a second translation team. The accuracy of the translation was determined by comparing the second team’s version to the original text. However, as literature [41], this technique has limitations and requires translators to be proficient in both languages and familiar with both cultures. In the second phase, the instrument’s psychometric properties were analyzed to determine its validity, and translations were evaluated based on the similarity between the translated and original texts.

To overcome this situation, the researchers sought the assistance of three translators to ensure the accuracy and consistency of the scale’s interpretation and presentation. The translators worked independently, and the authors and translators agreed on the final version. An English professor later modified the scale for clarity and accessibility to the public, while maintaining the original item length as much as possible. In addition to the escapism scale, the questionnaire also included sociodemographic questions about age group, principal, educational status, father’s education, mother’s education, smoking by parents, and occupational status of fathers and mothers. The second phase of the study involved testing the escapism scale for reliability, validity, and confirmatory factor analysis. The scale’s construct validity was assessed through concurrent validity, as well as Cronbach’s alpha and test-retest reliability [42]. The second step involved collecting data from a fresh sample of Iranian adolescents through Google Forms. As participants had the freedom to choose which items to answer, no data was missing. We calculated the CFA using the new sample.

### Measures

The researchers used a demographic characteristics checklist to collect information on several variables, including age group, major, educational status, father’s education, mother’s education, smoking habits of parents, and occupational status of both fathers and mothers.

#### The escapism scale

A self-report measure was used to assess self-expansion and self-suppression through activity engagement, adapted from Stenseng et al., [14]. The scale comprises 11 items, with the first five measuring self-expansion, the six item measuring self-suppression. Participants rate each item using a 7-point Likert scale, ranging from one (strongly disagree) to seven (strongly agree). The scores for self-expansion and self-suppression are calculated separately by adding the ratings for their respective items, with higher scores indicating greater levels of each dimension. A confirmatory factor analysis (CFA) was performed and indicated that the scale demonstrated a satisfactory fit with the data: χ² (40, N = 207) = 90.48, CFI = 0.95, NFI = 0.91, and RMSEA = 0.073. All factor loadings were above 0.44 for all dimensions [28].

#### Erikson psychosocial stage inventory (EPSI)

A subscale of this inventory measures adolescent identity (identity confusion, six items; identity coherence, six items) [43]. This inventory measures adolescents’ feelings about themselves and their beliefs. The EPSI has been developed for use with adolescents and adults. Identity coherence and identity confusion are two subscales. Identity consistency was reported to have an alpha coefficient of 0.80, while identity confusion was reported to have an alpha coefficient of 0.70 [43]. Reis and Youniss [44] obtained 0.82 Cronbach’s alpha for identity consistency and 0.79 for identity confusion in their study.

We used the **Satisfaction with Life Scale (SWLS)** to assess participants’ level of life satisfaction [21]. The survey consists of several items, and participants rate their level of agreement on a Likert scale from 1 (strongly disagree) to 7 (strongly agree). The total score ranges from five to 35, with higher scores indicating higher levels of life satisfaction. The reliability of this scale using Cronbach’s alpha formula was reported as 0.87 [45].

#### The smartphone addiction scale-short version (SAS-SV)

To evaluate the validity and reliability of the Persian version of the Escapism Scale, Iranian adolescents aged 14–18 were recruited. The Smartphone Addiction Scale (SAS), initially developed by Kim, Cho, and Yang in 2013, was used to develop a short version of the scale (SAS-SV) specifically for adolescents in 2013 [46]. Based on a Likert scale of 1 to 6, I have rated this scale on a scale of 1 (“completely disagree”) to 6 (“completely agree”) [47]. The original study obtained a Cronbach’s alpha coefficient of 0.91, and the present study had a Cronbach’s alpha coefficient of 0.82.

We used the **Hope Scale** [48]. Based on 12 items, the survey assesses a respondent’s level of hope for the future. Snyder’s cognitive model of hope is divided into two subscales: (1) Agency, which measures goal-directed energy, and (2) Pathways, which measures planning to reach goals. Responses are rated on a Likert-type scale, ranging from " false” to “true“ [49]. Four items on this scale are fillers. Nooripour et al. [50] reported 0.79 for its Cronbach’s alpha. The reliability of the study was measured by Cronbach’s alpha of 0.82.

#### General self-efficacy (GSE)

The General Self-Efficacy Scale was developed by Schwarzer and Jerusalem in 1979 and revised to include ten items in 1981, all of which generally measure self-efficacy. Scoring is based on a four-point Likert scale ranging from one to four. The Cronbach’s alpha coefficient for this scale was 0.82, indicating good internal consistency [51]. In Iran, Cronbach’s alpha coefficient of this scale was 0.81 [52].

### Data analysis

We conducted a correlation analysis between the Escapism Scale and identity styles, hope, satisfaction with life, general self-efficacy, and smartphone addiction using IBM SPSS Statistics 26.0 (IBM SPSS Statistics, Inc., Armonk, USA). To determine the internal consistency of the Escapism Scale, we used Cronbach’s alpha. Additionally, we employed a CFA model using LISREL 8.8 to develop the two-factor structure of the Escapism Scale. Our analysis was based on several fit indices, including the Root Mean Square Error of Approximation (RMSEA), the Parsimony Normed Fit Index (PNFI), the Comparative Fit Index (CFI), the Incremental Fit Index (IFI), the Standardized Root Mean Square Residual (SRMR), and the Normed Fit Index (NFI). It is recommended that the CFI, IFI, and NFI be above 0.90, the AGFI be greater than 0.80, the PNFI be greater than 0.50, the RMSEA remain below 0.08, and the SRMR not exceed 0.09. We also calculated the Cronbach’s alpha coefficient.

### Preliminary analysis

To prepare for the CFA, we conducted preliminary tests to analyze the data loss, discarded data, and normality of the data. We used AMOS software to identify scrapped data using Mahalanobis distance squared at a significance level of 0.001. Additionally, we tested the normality assumption using skewness (0.154–0.181) and kurtosis (0.35–2.15). Actually, according to Ryu [53], skewness scores between − 2 and 2, and kurtosis scores between − 7 and 7 indicate a normal distribution of data. This means that if the skewness score is outside the range of -2 to 2 or the kurtosis score is outside the range of -7 to 7, then the data may not be normally distributed.

## Results

A total of 566 questionnaires were analyzed for this study, with participants ranging in age from 14 to 18 years. Of the 566 adolescents who took part, 340 (60.1%) were girls and 226 (39.9%) were boys. The majors of the participants were as follows: 50 (8.8%) technical, 130 (23.0%) work and knowledge, 134 (23.7%) humanities, 186 (32.9%) experimental and 66 (11.7%) mathematics. In terms of fathers’ education, 28 (4.9%) were illiterate, 134 (23.7%) had primary education, 102 (18.0%) had secondary education, 164 (29.0%) had a diploma, 42 (7.4%) were graduates, 58 (10.2%) had a bachelor’s degree, 18 (2.3%) had a graduate degree, and 20 (3.5%) did not provide an answer. For mothers’ education, 64 (11.3%) were illiterate, 186 (32.9%) had primary education, 108 (19.1%) had secondary education, 134 (23.7%) had a diploma, 34 (6%) were graduates, 36 (6.4%) had a bachelor’s degree, 2 (0.4%) had a master’s degree or higher, and 2 (0.4%) did not provide an answer. A total of 174 fathers (30.7%) and 6 mothers (1.1%) were smokers. The employment status of fathers was as follows: 6 (1.1%) were unemployed, 352 (62.2%) were self-employed, 120 (21.2%) were in government jobs, 64 (11.3%) were retired, and 24 (4.2%) did not provide an answer. For mothers’ employment status, 508 (89.8%) were homemakers, 8 (1.4%) were self-employed, 32 (5.7%) were in government jobs, 14 (2.5%) were retired, and 4 (0.7%) did not provide an answer.

### Factor structure

**Fig. 1:**
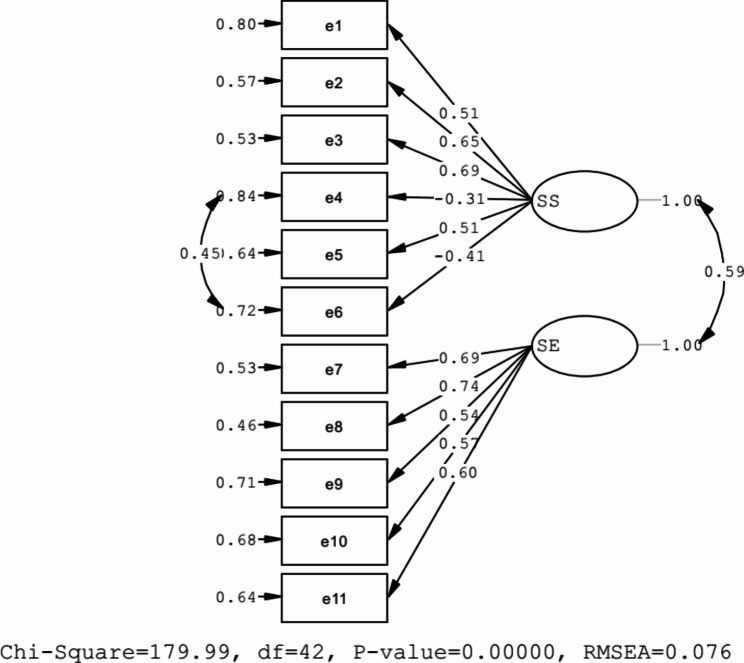
Model fit indexes of escapism scale among Iranian adolescents

Based on the findings, all items showed statistically significant factor loadings, with factor loading for all items being over 0.40 except for item 4, which was 0.31 (refer to Table 1). Therefore, it may be advisable to omit item 4. The results indicate that the model fits well with the data, as evidenced by the examination of the fit of the studied model. Furthermore, the results support the two-factor model, as shown in Table 2; Fig. 1.

**Table 1:** Factor loadings and descriptive indices for the items of the escapism scale

		Items statistics			Item-Total statistics		
Items	Component	Mean	SD	Factor Loading	V	I.T.	C.D.
1	Self-Suppression	1.95	0.878	0.51	14.368	0.213	0.677
2	Self-Suppression	1.84	0.803	0.65	13.338	0.338	0.637
3	Self-Suppression	2.08	0.889	0.69	12.803	0.419	0.626
4	Self-Suppression	2.86	0.942	0.31	16.393	-0.134	0.761
5	Self-Suppression	2.15	0.871	0.51	13.043	0.391	0.634
6	Self-Suppression	2.99	0.893	0.41	16.751	-0.176	0.764
7	Self-Expansion	1.68	0.667	0.69	13.734	0.421	0.638
8	Self-Expansion	1.63	0.684	0.74	13.789	0.394	0.642
9	Self-Expansion	2.28	0.878	0.54	12.836	0.422	0.626
10	Self-Expansion	1.88	0.787	0.57	13.836	0.310	0.655
11	Self-Expansion	2.04	0.855	0.60	12.980	0.413	0.629

***Note***: **V** = scale variance if item deleted, **I.T.** = corrected item-total correlations, **C.D.** = Cronbach’s alpha if item deleted

**Table 2:** CFA and Fit indexes

Model	RMSEA (CI 90%)	_sb_X^2^	RMR	SRMR	CFI	NFI	IFI	AGFI	GFI
BM	0.12 (0.11–0.13)	389.78	0.086	0.066	0.84	0.84	0.85	0.83	0.89
AM	0.076 (0.068–0.085)	179.99	0.056	0.07	0.91	0.89	0.91	0.90	0.93

***Legend***: BM = Before Modification, AM = After Modification, RMSEA: Root Mean Square Error of Approximation; RMR: Root Mean Square Residual; SRMR: Standardized RMR; CFI: Comparative Fit Index; NFI: Normed Fit Index; IFI: Incremental Fit Index; RFI: Relative Fit Index; AGFI: Adjusted Goodness of Fit Index; GFI: Goodness of Fit Index

The CFA was used to test the two-factor model, and it provided a marginal fit to the data. The findings for the two-factor structure are presented in Table 2. The results indicated that the two-factor model fit the data well: sbX2 = 179.99 (p < 0.01); SRMR = 0.07; RMR = 0.56, CFI = 0.91; NFI = 0.89; IFI = 0.91; NFI = 0.89; GFI = 0.93; AGFI = 0.90, RMSEA = 0.076. These results demonstrated that all standardized factor loadings for all items were statistically significant, supporting each item adequately for each component (p < 0.05). The factor loading for self-suppression items ranged from 0.31 to 0.69, and for self-expansion items, from 0.54 to 0.74 (Table 1). As presented in Table 1; Fig. 1, all items of loads show a significant factor and standardized factor loading for all items over 0.40 except item 4 (0.31).This item can potentially be omitted, but further investigation is necessary to determine its relevance. Without considering this issue, it can be concluded that the Persian version of the scale maintains the same items as the original version and keep total items.

### Internal consistency and test-retest reliability

The Farsi Version of the Escapism Scale demonstrated good internal consistency with a Cronbach’s alpha of 0.73, indicating that the items on the scale were measuring the same construct. The Guttman’s split-half coefficient of 0.61 and McDonald’s omega (ω) coefficient of 0.746 also suggest that the scale has acceptable reliability. Additionally, the test-retest coefficient of 0.87 after two weeks indicates good temporal stability, meaning that the scale produces consistent results over time. These findings support the use of the Farsi Version of the Escapism Scale in future research on escapism among Iranian adolescents (CI = 0.81–0.85).

### Concurrent validity

**Table 3:** Correlation between components of escapism and identity styles, hope, satisfaction with life, smartphone addiction, general self-efficacy

	Escapism (Total)	Self-Expansion	Self-Suppression
Identity Confusion	0.164**	-0.194**	0.048
Identity Coherence	-0.29**	-0.266**	-0.162**
AHS	-0.31**	-0.34**	-0.18*
SWLS	-0.34**	-0.39**	-0.21**
GSE	-0.33**	-0.35**	-0.24**
SAS-SV	0.19**	0.24**	0.09

** P < 0.001

***Note: AHS*** = Snyder’s Hope Scale; ***SWLS*** = Satisfaction with Life Scale; ***GSE*** = General Self-efficacy; ***SAS-SV*** = Smartphone Addiction Scale–Short Version

The results presented in Table 3 show that there is a correlation between the different components of escapism and various psychological variables in adolescents. Firstly, a significant positive relationship was found between the total score of escapism and identity confusion (r = 0.164, P < 0.01) as well as identity coherence (r = 0.29, P < 0.01). Self-expansion was also found to have a significant positive relationship with identity confusion (r = 0.194, P < 0.01) and a significant negative relationship with identity coherence (r=-0.266, P < 0.01). Self-suppression was found to have a significant negative relationship with identity coherence (r=-0.162, P < 0.01), but no significant relationship was observed between self-suppression and identity confusion (r = 0.048, P = 0.41). Furthermore, the findings showed that there was a significant negative relationship between escapism and AHS (r=-0.31, P < 0.01), SWLS (r=-0.34, P < 0.01), and GSE (r=-0.33, P < 0.01), indicating that as the level of escapism increased, the levels of these psychological variables decreased. On the other hand, there was a significant positive relationship between escapism and SAS-SV (r = 0.19, P < 0.01), indicating that as the level of escapism increased, the level of stress and anxiety also increased. Overall, these results suggest that the Farsi version of the Escapism Scale has acceptable concurrent validity, as shown in more detail in Table.

### Escapism and gender differences

The subscales of escapism in adolescent girls and boys were compared using the MANOVA test. The assumption of the equality of variance-covariance matrices was found to be not met, as indicated by the Box’s M Test (F(3, 2948071.38) = 3.73, P < 0.05). However, it should be noted that according to Tabachnick and Fidell [54], MANOVA is robust to this assumption when the sample size is large enough.

The Hotelling’s T-squared test results for the combination of escapism subscales revealed a significant group effect between adolescent girls and boys (F(2,53) = 8.05, P < 0.001, η2 = 0.028). To further elucidate the differences between the two groups in the subscales of escapism, Table 4 presents a detailed account of the results obtained using multivariate analysis of variance.

**Table 4:** Descriptive statistics of girl and boy groups in terms of subscales of Escapism

Subscales	Gender	Number	Mean	SD
Self-Suppression	Boy	340	14.17	2.59
	Girl	226	13.45	3.03
Self-Expansion	Boy	340	9.77	2.59
	Girl	226	9.12	2.96
Escapism Total	Boy	340	23.94	3.45
	Girl	226	22.58	4.64

**Fig. 2:**
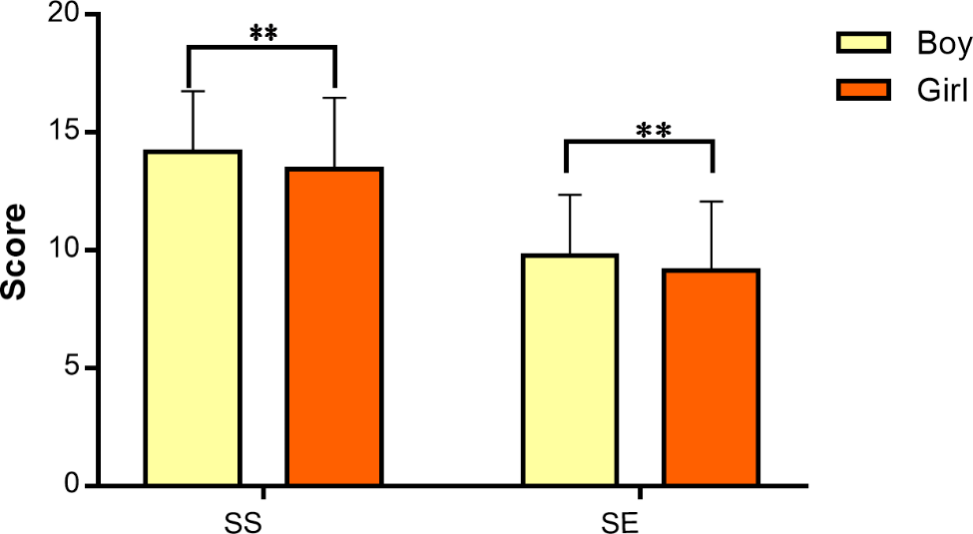
Gender differences in Escapism subscales. **Note** *(SS = Self-Suppression, SE = Self-Expansion)*

Based on the results of the univariate analysis of variance presented in Table 5, it can be inferred that there is a significant difference between the girl and boy groups in the self-suppression (F(1,564) = 6.67, P < 0.01) and self-expansion (F(1,564) = 4.86, P < 0.01) subscales (refer to Fig. 2).

**Table 5:** Multivariate Analysis of Variance Test for Boy and Girl groups of Escapism Scale

Source of Changes	Variable	Mean of Square	df	F	*P*	Effect size
Group	Self-Suppression	69.08	1-564	10.38	0.001	0.018
	Self-Expansion	56.77	1-564	7.53	0.006	0.013

## Discussion

The purpose of this research was to evaluate the psychometric properties of the Persian version of Escapism Scale among Iranian adolescents aged 14–18 years old regarding validity and reliability. Our results confirmed that the escapism scale was effective in assessing self-expansion and self-suppression among Iranian adolescents aged 14–18, with sufficient internal coherence. No items were removed before conducting the analysis, and two components were extracted from the scale using varimax rotation. Additionally, all of the item-wide associations were positive, indicating that all of the elements shared a common underlying characteristic.

This research aimed to gain a better understanding of escapism by utilizing a recently developed model. The dualistic model of escapism, which distinguishes between the dimensions of self-suppression and self-expansion, was used to examine escapism in terms of these two dimensions. Previous studies have established a correlation between these dimensions and positive and negative effects such as hope, self-efficacy, and smartphone addiction. The increasing prevalence of escapism highlights the need for further research. Psychology can benefit greatly from escapism research as it deals with both individual and social processes. Furthermore, this study explored the roles of self-suppression and self-expansion in escapism, two states that are somewhat opposed to each other. It is crucial to emphasize that these findings support the dual nature of escapism and encourage individuals to reflect on their attitudes and emotions regarding their participation in such activities [9]. The literature review did not yield extensive research on the validity of escapism and its relationship with identity, life satisfaction, smartphone addiction, hope, and self-efficacy. However, it is worth noting that escapism had varying associations with well-being when measured using global and activity-related indicators of well-being [55].

Escapism can be conceptualized in two dimensions in this study. The expansion of well-being and the suppression of ill-being are two dimensions of self-expansion. Escapism has two motives: self-expansion and self-suppression. We found both dimensions are associated with explanations for action attention. Self-suppression leads to poor psychological adjustment, while self-expansion leads to better psychological change. Escapism was also negatively correlated with hope, life satisfaction, and general self-efficacy. An association was found between activity engagement and better affective outcomes in the presence of escapism. Motivational dualities include self-expansion and self-suppression. It has been found that individuals engage in activities based on various motivational mindsets, which affect the outcome of the engagement. Studies should be conducted to examine the conditions that lead to the development of self-suppression and self-expansion. The purpose of escapist activities is to be instrumental. This is because they provide a break from stagnation, an opportunity to explore the unknown, and the chance to follow one’s imagination. In everyday life, people are often faced with demands for self-expansion; in the workplace, for example, employees are expected to adapt but also to evolve, transform, and think outside the box, leading to self-expansion escapism (gaining knowledge, becoming more aware of oneself, opening up to new experiences that would enhance one’s life) [14].

Considering the lack of positive emotions for escapism, researchers may want to explore client expectations regarding things that create happiness in their lives. There is a possibility that these clients are predisposed to negative mood states because of attention bias or cognitive distortions [56]. They may also be vulnerable to serial disappointments because of unrealistic expectations. A more detailed examination of this hypothesis is necessary.

The study provides valuable insights into the concept of escapism by presenting data and analyses from a non-clinical population, which can inform future research and suggest the need for testing in clinical populations. However, the current findings may not directly apply to clinical practice, since they need to provide specific recommendations for interventions or treatments. Nevertheless, the study’s findings can still be helpful for clinicians in several ways. Firstly, the study highlights the importance of recognizing escapism as a phenomenon that individuals used to cope with emotional distress, which can inform clinical assessments and help clinicians identify patients who may engage in such behaviors. Secondly, the study’s results indicate that escapism is associated with more unpleasant mood states, suggesting that it may not be an effective coping mechanism in the long term. Clinicians can use this information to help patients develop more adaptive coping strategies that address the underlying emotional issues causing distress. In summary, while the current findings may not have immediate applications, they can still provide valuable insights and inform future research on escapism. Clinicians can use this information to improve their assessments and develop more effective interventions for patients who engage in escapism behaviors.

The present study shows that the escapism scale can be used as a reliable tool to assess self-suppression and self-expansion in Iranian adolescents. Future studies should investigate whether these dimensions of escapism are specific to certain populations, such as adolescents, or whether they are applicable across different age groups and cultures. While this scale has been studied in various fields, its applicability to university students remains underexplored, leaving the analysis of behaviors associated with escapism incomplete, regardless of the sociocultural context. Therefore, future research could delve into the use of this scale among university students to gain a deeper understanding of escapism and its related behaviors. Although the escapism scale captured escapism in the present participants, it will be necessary to reproduce these results in both clinical and non-clinical populations at multiple developmental stages. Testing the escapism scale in more diverse samples of people from non-English-speaking countries is imperative.

### Limitations

The study’s limitations mainly relate to the sample, measurements, and analysis. Our sample included students from various social studies and natural science disciplines and ages. Future studies should provide more gender, age, and educational structure variability to increase generalizability. The measurement instruments used in this study were only self-assessment scales, so there is already a potential bias in the measurement. Interpreting conclusions beyond these findings with caution is essential because most participants were not seeking treatment. Research should investigate intrinsic and extrinsic motivations for escapism and personality traits associated with escapism, such as openness to experiences, fantasy-prone personalities, self-criticism, anxiety tendencies, and time perspective.

## Conclusion

After conducting the study, it was found that the 11-item escapism scale was valid and reliable for assessing escapism among Iranian adolescents. Therefore, psychologists, clinicians, and researchers who are working with this specific population can use this psychological assessment instrument. In addition, since the escapism scale has the capability to evaluate a wide range of escapism, it can be an effective tool for assessing escapism in Iranian adolescents. Thus, this instrument can be a valuable addition to the existing psychological assessment tools that are available to professionals who work with this population.

## Data Availability

The corresponding author will provide the datasets generated and analyzed during this study upon a reasonable request.
